# Photodeposition of Ag_2_S on TiO_2_ nanorod arrays for quantum dot-sensitized solar cells

**DOI:** 10.1186/1556-276X-8-10

**Published:** 2013-01-03

**Authors:** Hongwei Hu, Jianning Ding, Shuai Zhang, Yan Li, Li Bai, Ningyi Yuan

**Affiliations:** 1 Center for Low-Dimensional Materials, Micro-nano Devices and System, Changzhou University, Changzhou, 213164, China; 2 Jiangsu Key Laboratory for Solar Cell Materials and Technology, Changzhou, 213164, China

**Keywords:** Ag_2_S, Quantum dot-sensitized solar cell, Photodeposition, TiO_2_ nanorod

## Abstract

Ag_2_S quantum dots were deposited on the surface of TiO_2_ nanorod arrays by a two-step photodeposition. The prepared TiO_2_ nanorod arrays as well as the Ag_2_S deposited electrodes were characterized by X-ray diffraction, scanning electron microscope, and transmission electron microscope, suggesting a large coverage of Ag_2_S quantum dots on the ordered TiO_2_ nanorod arrays. UV–vis absorption spectra of Ag_2_S deposited electrodes show a broad absorption range of the visible light. The quantum dot-sensitized solar cells (QDSSCs) based on these electrodes were fabricated, and the photoelectrochemical properties were examined. A high photocurrent density of 10.25 mA/cm^2^ with a conversion efficiency of 0.98% at AM 1.5 solar light of 100 mW/cm^2^ was obtained with an optimal photodeposition time. The performance of the QDSSC at different incident light intensities was also investigated. The results display a better performance at a lower incident light level with a conversion efficiency of 1.25% at 47 mW/cm^2^.

## Background

Quantum dot-sensitized solar cells (QDSSCs) have attracted increasing attention due to their relatively low cost and potentials to construct high-efficiency energy conversion systems [1]. Compared with organic dyes used in dye-sensitized solar cells (DSSCs), semiconductor sensitizers in the form of quantum dots (QDs) present higher extinction coefficients and adjustable absorption spectra by controlling their size [2,3]. However, the best efficiency (approximately 5%) reached by QDSSCs is much lower than that of conventional DSSCs [4,5]. The deposition of QD sensitizers on the electron acceptor (e.g., TiO_2_) related to the loading amount and the connection between QDs and electron acceptor plays a key role in the QDSSC performance. QDs with various sizes should be deposited on the surface of mesoporous TiO_2_ separately as a requirement for efficient charge separation [6]. Typically, the coverage of mesoporous TiO_2_ by QDs is much less than a full monolayer [6,7], which leads to insufficient light harvesting and back electron transfer from exposed TiO_2_ to electrolyte. Besides, deposition of typically 3 to 8 nm diameter QDs into mesoporous TiO_2_ with relative narrow pores is rather difficult, and large QDs that inserted into mesoporous TiO_2_ may also cause pore blocking and subsequently inhibit the penetration of electrolyte deep into the holes [8]. The efficiency enhancement of QDSSCs could be achieved by applying an advanced deposition method as well as suitable TiO_2_ nanostructure. For the former, several deposition methods have been developed to anchor QDs on the surface of TiO_2_ including *ex-situ* and *in-situ* methods [6], where photodeposition is a promising candidate by taking advantage of the photocatalytic properties of TiO_2_ in the deposition process [9-11]. Photoreduction on the surface of TiO_2_ leads to a large and uniform coverage of QDs and intimate contact between the QDs and TiO_2_ for efficient interfacial charge transfer [11]. For the latter, one-dimensional oriented arrays (nanotube or nanorod arrays) possess large surface area and efficient electron transfer property that can be employed to improve the performance of QDSSCs [12,13]. Importantly, the high-oriented arrays provide uniform pore size that is favorable for QD anchoring with rare pore blocking.

Ag_2_S is an important photoelectric material and has a broad application in terms of photocatalysis and electronic devices [14-17]. With bulk bandgap of 1.0 eV, close to the optimal bandgap of 1.1 to 1.4 eV for photovoltaic devices [18], Ag_2_S is a potential sensitizer superior to others used in QDSSCs. Several researches that concentrated on the Ag_2_S-QDSSCs have been reported since the first application of Ag_2_S in QDSSCs [19-23]. However, the reported conversion efficiency (*η*) remains lower than that of QDSSCs based on other narrow bandgap semiconductor (e.g., CdS and CdSe) [24,25], which is partly attributed to the low coverage of Ag_2_S on the surface of TiO_2_.

To improve the efficiency of Ag_2_S-QDSSCs, we apply a modified photodeposition as well as an oriented TiO_2_ nanorod array (NRA) on the cell. Typically, the oriented TiO_2_ NRA was prepared by a simple hydrothermal method. Photodeposition of Ag_2_S was conducted by two steps: photoreduction of Ag^+^ to Ag by TiO_2_ NRA followed by the sulfurization of Ag to Ag_2_S QDs. To our knowledge, this is the first report of Ag_2_S QD-sensitized TiO_2_ NRA solar cells. Results show that a large coverage of Ag_2_S QDs on the TiO_2_ NRs has been achieved by this modified photodeposition, and the photoelectrochemical properties of these electrodes suggest that Ag_2_S has a great potential for the improvement of QDSSCs.

## Methods

### Growth of TiO_2_ NRA

TiO_2_ NRA was grown on the fluorine-doped SnO_2_-coated conducting glass (FTO) substrate (resistance 25 Ω/square, transmittance 85%) by a hydrothermal method as described in the literature [26]. Briefly, 30 mL deionized water was mixed with 30 mL concentrated hydrochloric acid (36.5% to 38.0% by weight). The mixture was stirred for 5 min followed by an addition of 1 mL titanium butoxide (98%, Sinopharm Chemical Reagent Co. Ltd., Shanghai, China). After stirring for another 5 min, the mixture was transferred into a Teflon-lined stainless steel autoclave of 100-mL volume. The FTO substrate was placed at an angle against the wall of the Teflonliner with the conducting side facing down. After a hydrothermal treatment at 150°C for 20 h, the substrate was taken out and immersed in 40 mM TiCl_4_ aqueous solution for 30 min at 70°C. The TiCl_4_-treated sample was annealed at 450°C for 30 min.

### Photodeposition of Ag_2_S on TiO_2_ NRA

**Figure 1:**
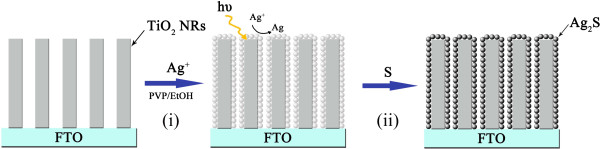
**Schematic illustration of the deposition of Ag_**2**_S on TiO_**2**_ NRA.** (i) Photoreduction of Ag^+^ to Ag; (ii) sulfurization.

As illustrated in Figure 1, the photodeposition procedure was conducted in two steps. Firstly, the as-prepared TiO_2_ NRA was immersed into the ethanol solution containing Ag^+^. The solution was prepared by dissolving 0.2 g polyvinylpyrrolidone (K90, MW = 1,300,000, Aladdin Chemical Co., Ltd., Shanghai, China) in 20 mL pure ethanol, followed by adding 0.2 mL of AgNO_3_ aqueous solution (0.1 M) dropwise. Irradiation was carried out from the direction of TiO_2_ film with a high-intensity mercury lamp for a given period. After irradiation, the substrate was taken out, washed with ethanol, and transferred into methanol solution consisting 1 M Na_2_S and 2 M S. The sulfurization reaction was conducted at 50°C for 8 h. Finally, the photoanodes were passivated with ZnS by dipping into 0.1 M Zn(CH_3_COO)_2_ and 0.1 M Na_2_S aqueous solution for 1 min alternately.

### Solar cell assembly

The counter electrode was prepared by dripping a drop of 10 mM H_2_PtCl_6_ (99.99%, Aldrich Company, Inc., Wyoming, USA) ethanol solution onto FTO substrate, followed by heating at 450°C for 15 min. Ag_2_S-sensitized TiO_2_ nanorod (NR) photoanode and Pt counter electrode were assembled into sandwichstructure using a sheet of a thermoplastic frame (25-μm thick; Surlyn, DuPont, Wilmington, USA) as spacer between the two electrodes. The polysulfide electrolyte consisted of 0.5 M Na_2_S, 2 M S, 0.2 M KCl, and 0.5 M NaOH in methanol/water (7:3 *v*/*v*). An opaque mask with an aperture was coated on the cell to ensure the illuminated area of 0.16 cm^2^.

### Characterization

X-ray diffraction (XRD) measurements were carried out using a RAD-3X (Rigaku Corporation, Tokyo, Japan) diffractometer with Cu-Kα radiation. The morphology of the films was observed by field emission scanning electron microscopy (FESEM, S4800, Hitachi Ltd., Tokyo, Japan) and transmission electron microscope (TEM, JEM-2100, JEOL Ltd., Beijing, China). To prepare the TEM sample, TiO_2_ NRs together with Ag_2_S QDs were scratched from the FTO substrate and dispersed in ethanol by sonication. The UV–vis absorption spectra of TiO_2_ NRA and Ag_2_S-deposited TiO_2_ NRA were recorded in the range from 350 to 800 nm using a Hitachi U-3010 spectroscopy. The photocurrent density-voltage (*J*-*V*) characteristics of solar cells were examined by a Keithley 2400 sourcemeter (Keithley Instruments, Inc., Cleveland, USA) under illumination by a solar simulator (AM 1.5 G). Incident light intensity was calibrated by standard silicon solar cell and light intensity meter (FZ-Aradiometer) simultaneously. The stability of the solar cell was measured by electrochemical workstation (pp211; Zahner, Elektrik GmbH & Co.KG, Kronach, Germany) with continuous illumination on the solar cell.

## Results and discussion

### Morphology of the TiO_2_ NRA

**Figure 2:**
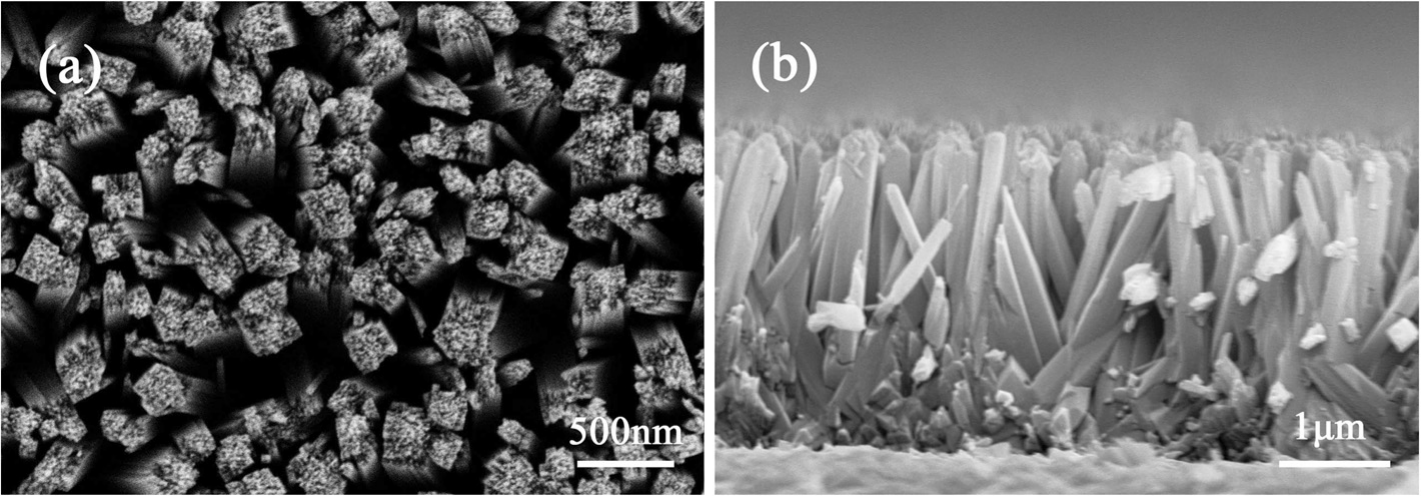
**FESEM images of TiO_**2 **_NRA.** Top (**a**) and cross-sectional views (**b**).

Figure 2 shows the FESEM images of TiO_2_ NRA grown on the FTO substrate (FTO/TiO_2_) viewed from top (a) and cross-section (b). The TiO_2_ film is composed of separate NRs with consistent orientation, forming a uniform array that covered the entire surface of the substrate. The top view of FTO/TiO_2_ shows that the top surface of NRs contains many step edges facilitating further growth. The NRs are tetragonal in shape with square top facets, consistent with the growth habit of tetragonal crystal structure. The average side length of the top squares is 200 nm, and the space between them is about the same size. The cross-section view of FTO/TiO_2_ shows that the NRs are 2 to 3 μm in length with smooth sides. At the bottom of the TiO_2_ NRA, a thin layer composed of short disordered NRs adhering to the FTO substrate is found. The compact layer may reduce the recombination of electron from the FTO to the electrolyte in the working course of QDSSCs by segregating them.

### Photodeposition of Ag_2_S QDs

**Figure 3:**
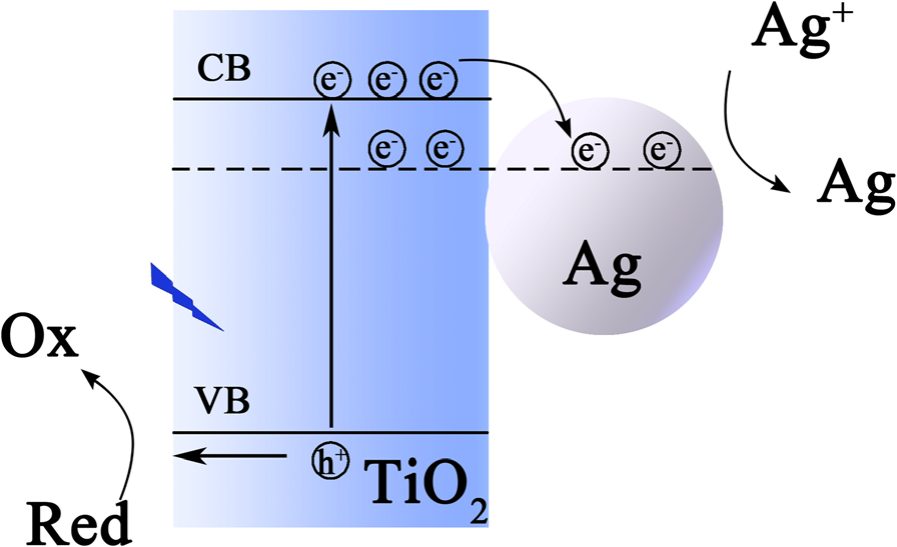
Schematic illustration for charge separation between TiO_**2 **_and Ag, and redox reaction on them.

The photodeposition of Ag_2_S QDs was conducted by two separate processes: photoreduction of Ag^+^ to Ag and sulfurization of Ag to Ag_2_S. Photocatalytic properties of TiO_2_ play an essential role in the reduction of Ag^+^. The mechanism of TiO_2_ photocatalytic-reduction metal ions was described in the literature [27]. The main reaction processes of photoreduction Ag^+^ are as follows (reactions 1 to 4): (1) Typically, TiO_2_ surface exhibits strong adsorptivity for Ag^+^, and the adsorption equilibrium is reached soon after immersing FTO/TiO_2_ in Ag^+^ ethanol solution in the dark. (2) UV irradiation (*λ* < 400 nm) excites TiO_2_ to generate electron–hole pairs. (3) The electrons reduce the Ag^+^ adsorbed preferentially on the surface to Ag, (4) while the holes are irreversibly scavenged by ethanol. Continuous reduction of Ag^+^ can produce Ag nucleates on the surface of TiO_2_ forming a Schottky junction between them. The charge-separation generated electrons are partially transferred to the Ag clusters from TiO_2_[28]. Oxidation and reduction processes are carried on at the surface of TiO_2_ and Ag, respectively, as illustrated in Figure 3. Consequently, the reduction on the surface of Ag enables the crystal nucleus to grow up. After the photoreduction, the sulfurization reaction of Ag clusters occurs spontaneously, owing to the low reaction Gibbs energy of −47.1 kJ/mol [29].

(1) $${\mathrm{TiO}}_{2}{+\mathrm{Ag}}^{+}\to {\mathrm{TiO}}_{2}{\mathrm{Ag}}^{+}$$

(2) $${\mathrm{TiO}}_{2}+\mathrm{hv}\to {\mathrm{TiO}}_{2}\left(e^{-}+h^{+}\right)$$

(3) $$e^{-}+{\mathrm{Ag}}^{+}\to \mathrm{Ag}$$

(4) $$h^{+}+C_{2}H_{5}\mathrm{OH}\to C_{2}H_{4}\mathrm{OH}$$

Photoreduction rate of Ag^+^ by TiO_2_ in ethanol solution is so rapid that the electrode turned to silvery-white within 3 min after immersing FTO/TiO_2_ in the solution. To verify the effect of photocatalytic properties of TiO_2_ on the reduction process, the ethanol solution containing Ag^+^ was irradiated in the same condition but in the absence of TiO_2_, and no silver was observed in 10 h. Similar results were also observed when immersing FTO/TiO_2_ in the Ag^+^ solution in the dark, consistent with the proposed photoreduction mechanism.

**Figure 4:**
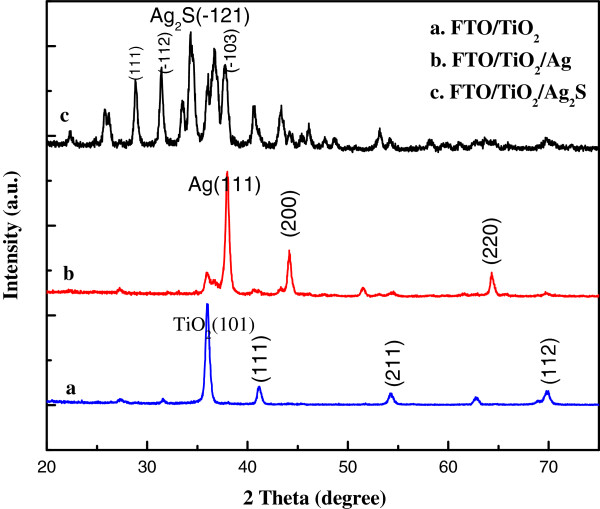
**XRD patterns.** FTO/TiO_2_ (a), FTO/TiO_2_/Ag (b), and FTO/TiO_2_/Ag_2_S (c) electrodes.

Figure 4 shows XRD patterns of FTO/TiO_2_ (a), FTO/TiO_2_/Ag (b), and FTO/TiO_2_/Ag_2_S (c) electrodes. XRD patterns of FTO/TiO_2_ electrode reveal that the synthesized TiO_2_ NRs are tetragonal rutile structure (JCPDS card no. 21–1276). The enhanced (101) peak indicates the NRs are well-crystallized and grow in consistent orientation. In the XRD pattern of FTO/TiO_2_/Ag electrode (b), all peaks indexed as TiO_2_ crystal have been weakened while the outstanding diffraction peaks of silver (silver-3C, syn JCPDS card no. 04–0783) emerged. This proves the large coverage of crystallized Ag on the surface of TiO_2_ nanostructure as a result of the photoreduction process. As compared with curve b, the XRD pattern of FTO/TiO_2_/Ag_2_S electrode shows five diffraction peaks which agreed well with acanthite Ag_2_S (JCPDS card no. 14–0072), suggesting a conversion of Ag to Ag_2_S. Additionally, the outstanding peaks of Ag in curve b are not observed in curve c which indicates that the reaction between Ag and S has been completed thoroughly.

**Figure 5:**
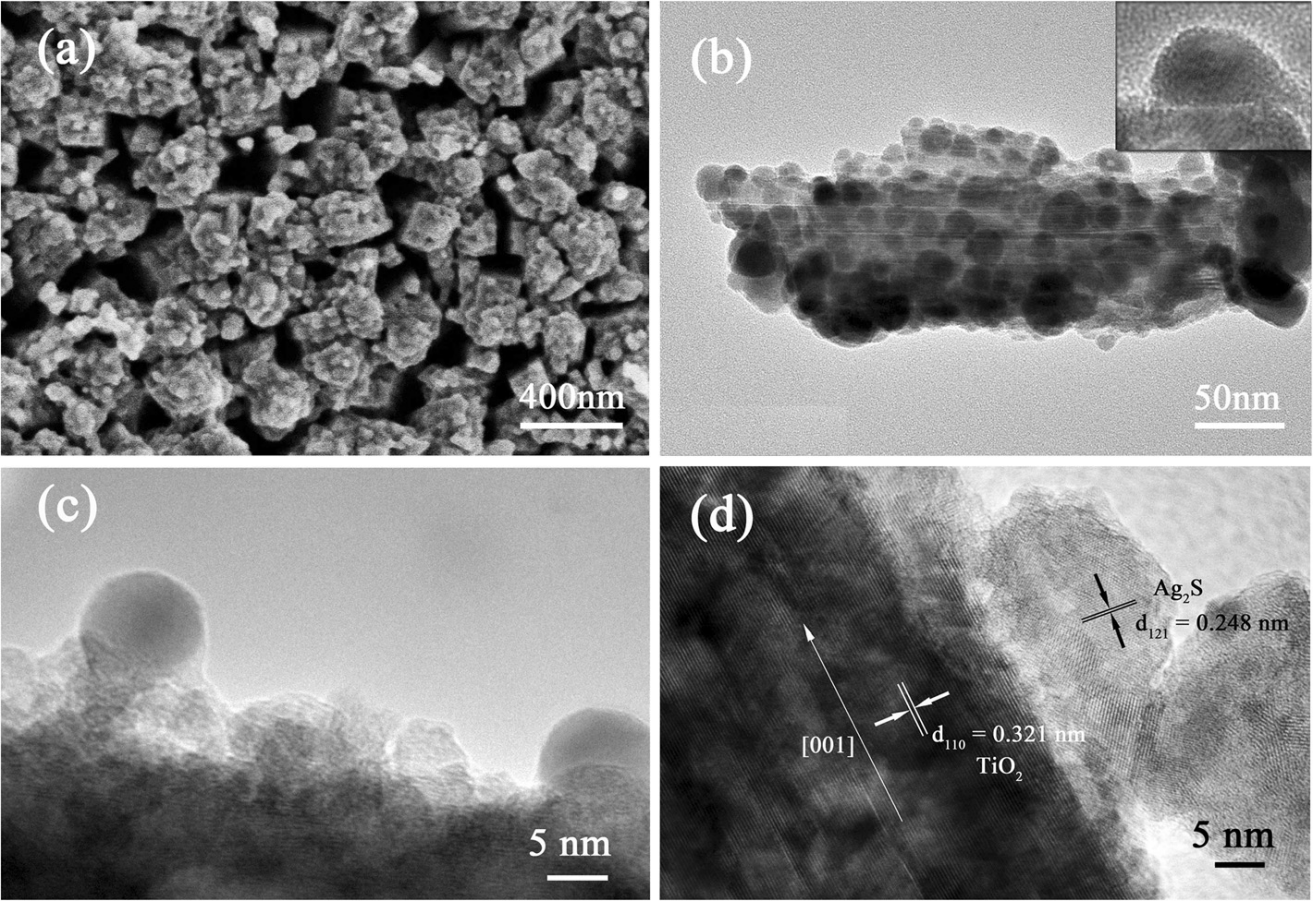
**SEM, TEM, and HRTEM images.** SEM image of FTO/TiO_2_/Ag_2_S (top view) (**a**), TEM image of a single TiO_2_ NR covered with Ag_2_S QDs (**b**), and HRTEM images of TiO_2_/Ag_2_S (**c**,**d**).

Figure 5 displays a SEM image of a top view of FTO/TiO_2_/Ag_2_S electrode with 10-min photoreduction (a) and a TEM image of single NR stripped from the FTO/TiO_2_/Ag_2_S electrode (b). The two images clearly show that TiO_2_ NRs are coated by a layer of Ag_2_S crystallites not only on the top surface but also on the four side faces. The top view of FTO/TiO_2_/Ag_2_S electrode shows that the small steps within the top face of TiO_2_ NR observed in SEM image of FTO/TiO_2_ electrode (Figure 2a) are invisible due to the coverage of Ag_2_S crystallites. The interval between NRs is reserved as well as the ordered TiO_2_ NRA structure. The TEM image (b) shows that the entire NR is coated with QDs from the bottom to the top. Most of the QDs that covered the surface of NR disperse well with an average diameter of 10 nm. A closer observation of the Ag_2_S QDs attached with TiO_2_ NR can be obtained by the high resolution transmission electron microscope (HRTEM) images (Figure 5c,d). The NR grows along the [001] direction, and lattice fringes with interplanar spacing *d*_110_ = 0.321 nm are clearly imaged. The Ag_2_S QDs anchoring on the side surface of TiO_2_ NR are composed of small crystallites as observed by the fringes which correspond to the (121) planes of Ag_2_S.

### Optical and photoelectrochemical properties of Ag_2_S QDs-sensitized TiO_2_ NRA

**Figure 6:**
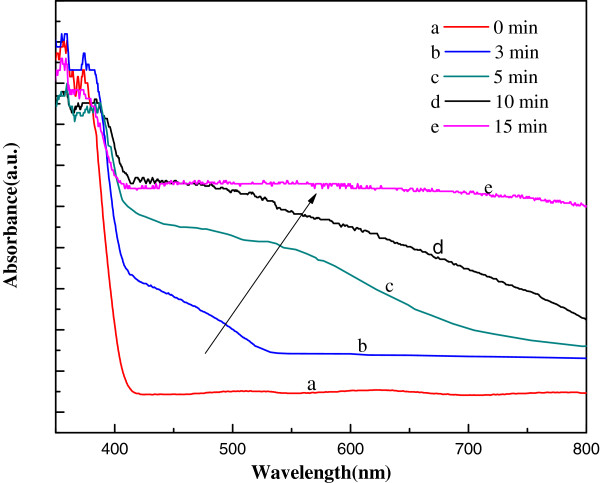
UV–vis absorption spectra of FTO/TiO_**2 **_electrode (a) and FTO/TiO_**2**_**/Ag**_**2**_S electrodes with different photoreduction times (b, c, d, e).

Figure 6 shows the absorption spectra of FTO/TiO_2_ electrode and FTO/TiO_2_/Ag_2_S electrodes with different photoreduction times (*t*_p_). The absorption edge around 400 nm is consistent with bandgap of rutile TiO_2_ (3.0 eV). While Ag_2_S QDs are deposited on TiO_2_ NRs, absorption spectra are successfully extended to visible wavelength. With *t*_p_ increasing from 3 to 15 min, the absorption range changes from 400 to 520 nm until covering the entire visible spectrum; moreover, the absorbance obviously increases. The bandgap of bulk Ag_2_S is 1.0 eV. The redshift of absorption edge for FTO/TiO_2_/Ag_2_S electrodes with prolonged *t*_p_ indicates the fact that the size of Ag_2_S QDs gradually increases, and the quantization effect of ultrasmall QDs gradually vanishes. The enhanced absorbance is due to the increased amount of deposited Ag_2_S QDs.

**Figure 7:**
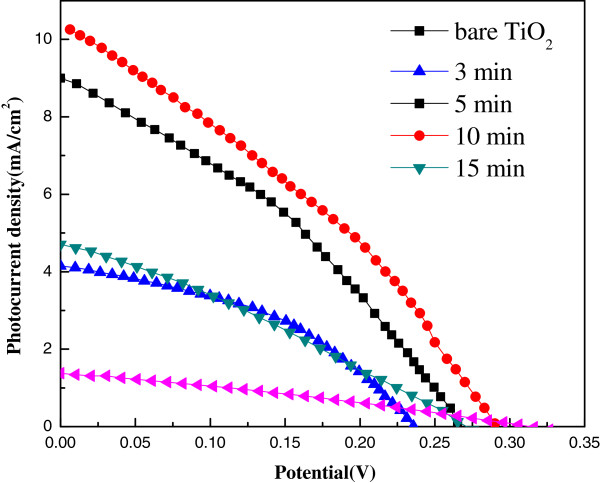
*J*-*V *characteristics of solar cells fabricated with different photoanodes under AM 1.5 illumination at 100 mW/cm^**2**^.

Figure 7 shows *J*-*V* characteristics of solar cells fabricated with different photoanodes under AM 1.5 illumination at 100 mW/cm^2^. The photovoltaic properties of these cells are listed in Table 1. TiO_2_/Ag_2_S cell with *t*_p_ = 3 min possesses a much higher *J*_sc_ and a decreased *V*_oc_ compared with bare TiO_2_ solar cell. The increased *J*_sc_ value is attributed to the sensitization of TiO_2_ by Ag_2_S QDs, while the slightly decreased *V*_oc_ value is mainly due to the band bending between Ag_2_S QDs and TiO_2_. With *t*_p_ increasing from 3 to 10 min, the *J*_sc_ is promoted from 4.15 to 10.25 mA/cm^2^. The improved *J*_sc_ value is caused by an increasing loading amount of Ag_2_S QDs and a broaden absorption spectrum (as shown in Figure 6). Meanwhile, the *V*_oc_ values are slightly improved, which is probably due to electron accumulation within TiO_2_ shifting the Fermi level to more negative potentials. The optimal solar cell performance is obtained with a *η* of 0.98% and a superior *J*_sc_ of 10.25 mA/cm^2^ when *t*_p_ = 10 min. The *J*_sc_ value is much higher than those of other reported Ag_2_S QD sensitized solar cells even though they were prepared by a TiO_2_ nanoparticle matrix with a larger surface area. The enhancement in *J*_sc_ is a result of the synergy of larger QD loading amount and fine connection between QDs and TiO_2_. Compared with typical QDSSCs based on other narrow bandgap semiconductors (e.g., CdS and CdSe), the *V*_oc_ values of Ag_2_S-QDSSCs are quite low which are almost equivalent to half of the others (CdS-QDSSCs, 0.6 to 0.7 V). Despite of the high *J*_sc_ values owing to a broad absorption spectrum, *η* is limited by the low *V*_oc_ values. When *t*_p_ was elongated to 15 min, *η* decreases sharply with a halving *J*_sc_ and a lower Fill factor (FF). This phenomenon is speculated to be caused by too long deposition time which results in excess Ag_2_S nanoparticles generated on TiO_2_ NRs, consequently decreases effective electron injection and increases recombination rate. The slightly reduced FF as *t*_p_ increases also indicates that recombination rate rises with growing amount of loading Ag_2_S nanoparticles.

**Table 1:** Photovoltaic parameters of solar cells fabricated with different photoanodes under AM 1.5 illumination at 100 mW/cm^**2**^

**Solar cell**	***J***_**sc**_**(mA/cm**^**2**^**)**	***V***_**oc**_**(V)**	**FF**	***η *(%)**
Bare TiO_2_	1.34	0.32	0.30	0.13
3 min	4.15	0.24	0.42	0.41
5 min	9.00	0.27	0.38	0.83
10 min	10.25	0.29	0.32	0.98
15 min	4.71	0.28	0.29	0.38

**Figure 8:**
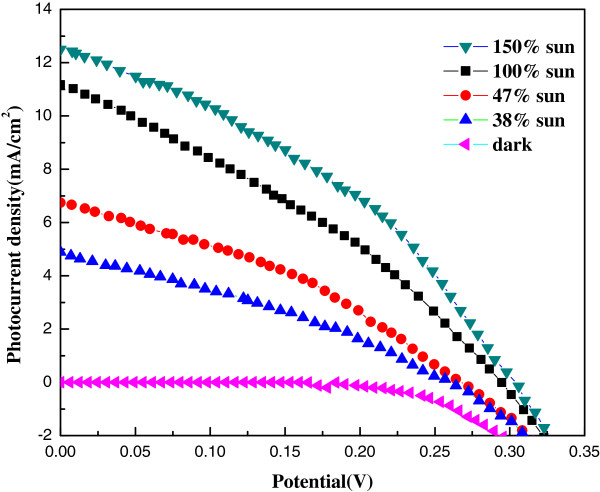
*J*-*V *curves of Ag_**2**_S QD-sensitized solar cell measured at different light intensities.

The *J*-*V* curves of a Ag_2_S QD-sensitized solar cell measured at three different light intensities are shown in Figure 8. The photovoltaic performance parameters are listed in Table 2. The *η* reaches a value of 1.25% at 47 mW/cm^2^ solar intensity. The *J*_sc_ value accumulates to 11.7 mA/cm^2^ as incident light intensity increases to 150 mW/cm^2^ (150% sun). However, *J*_sc_ produced by per unit light power is decreased by a factor of 40.9 compared with lower light level condition of 47% sun. This suggests that the incident light is not effectively converted into electricity at a higher photon density, which may be attributed to a lower rate of photon capture due to the insufficient QDs loading on TiO_2_ nanorods. By employing longer TiO_2_ NRs, the response of the photocurrent should be promoted to be linear with the incident light intensity, and a higher conversion efficiency should be reached at full sunlight.

**Table 2:** Photovoltaic parameters of Ag_**2**_S QD-sensitized solar cell measured at different light intensities

***P***_**in**_**(mW/cm**^**2**^**)**	***J***_**sc**_**(mA/cm**^**2**^**)**	***V***_**oc**_**(V)**	**FF**	***η *(%)**
150	11.7	0.3	0.37	0.87
100	10.3	0.29	0.33	0.98
47	6.2	0.26	0.36	1.23
38	4.6	0.25	0.32	0.97

**Figure 9:**
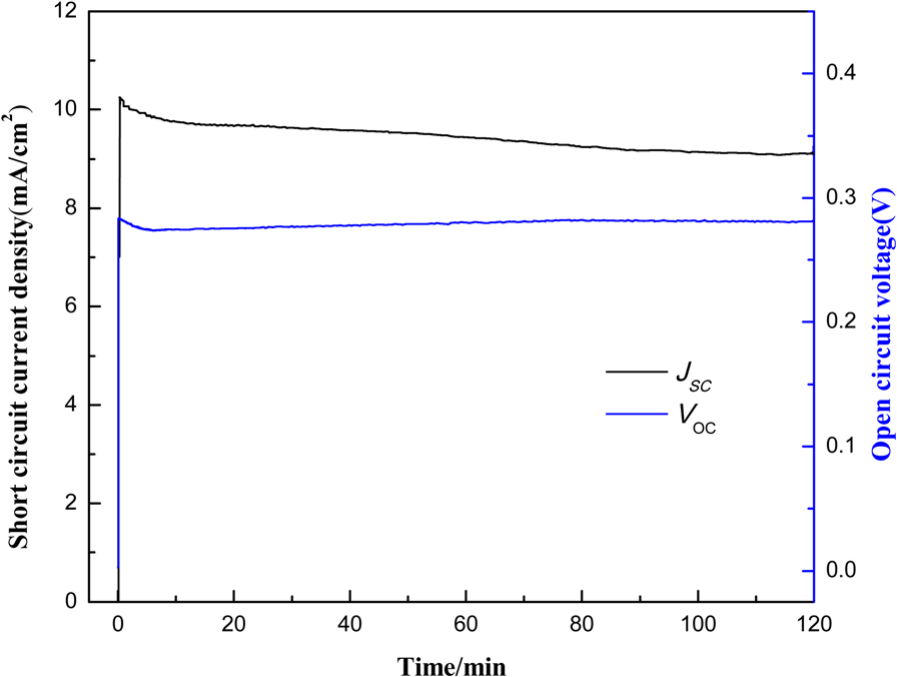
Photostability of Ag_**2**_S QD-sensitized solar cell under AM 1.5 illumination at 100 mW/cm^**2**^.

The photostability of Ag_2_S-QDSSC was measured by illuminating it at 100 mW/cm^2^ sunlight for 2 h and characterized by recording the *J*_sc_ and *V*_oc_ of the device (Figure 9). During illumination, the *J*_sc_ remained relatively steady with a drop less than 5%, and the *V*_oc_ fluctuated within 2%. This shows that the Ag_2_S QDs are robust in resisting photo corrosion in the presence of polysulfide electrolyte, and the small decline is probably caused by the loss of the solvent.

## Conclusions

We have deposited Ag_2_S QDs on TiO_2_ NRA by a two-step photodeposition. The deposition process was conducted by photoreduction of Ag^+^ to Ag on the surface of TiO_2_ NRs followed by chemical reaction with sulfur. By controlling the photoreduction period, we have obtained Ag_2_S-sensitized TiO_2_ NRs with a large coverage and superior photoelectrochemical properties. QDSSCs based on the Ag_2_S-sensitized TiO_2_ NRAs were fabricated. Under optimal condition, the Ag_2_S-QDSSC yields a *J*_sc_ of 10.25 mA/cm^2^ with a conversion efficiency of 0.98% at AM 1.5 solar light of 100 mW/cm^2^. We also investigated the solar cell performance under varied incident light intensities. Results show that a drawback of these cells in full sun condition compared with the maximum efficiency achieved at lower light level. The key factor that limits the solar cell performance is the low *V*_oc_ values we obtained. By employing suitable redox electrolyte, we believe the Ag_2_S-QDSSCs will have a great promotion with increased *V*_oc_ values.

## Abbreviations

η: conversion efficiency; DSSCs: dye-sensitized solar cells; FESEM: field emission scanning electron microscopy; FF: fill factor; FTO: fluorine-doped SnO_2_-coated conducting glass; HRTEM: high resolution transmission electron microscope; J_sc_: short circuit current density; NR: nanorod; NRA: nanorod array; QDs: quantum dots; QDSSCs: quantum dot-sensitized solar cells; SEM: scanning electron microscope; TEM: transmission electron microscope; t_p_: photoreduction time; V_oc_: open voltage; XRD: X-ray diffraction.

## Competing interests

The authors declare that they have no competing interests.

## Authors’ contributions

HWH carried out the experiments and wrote the manuscript. JND and NYY conceived the study, participated in its design, and amended the paper. SZ participated in the discussion and interpretation of the data. YL and LB participated in the experiments. All authors read and approved the final manuscript.
